# A novel partnership between lncTCF7 and SND1 regulates the expression of the TCF7 gene via recruitment of the SWI/SNF complex

**DOI:** 10.1038/s41598-024-69792-8

**Published:** 2024-08-21

**Authors:** Allison Yankey, Mihyun Oh, Bo Lim Lee, Tisha Kalpesh Desai, Srinivas Somarowthu

**Affiliations:** 1 Graduate School of Biomedical Sciences and Professional Studies, College of Medicine, Drexel University, Philadelphia, PA USA; 2 Department of Biochemistry and Molecular Biology, College of Medicine, Drexel University, Philadelphia, PA USA

**Keywords:** RNA, Long non-coding RNAs, Non-coding RNAs

## Abstract

Long non-coding RNAs (lncRNAs) play key roles in cellular pathways and disease progression, yet their molecular mechanisms remain largely understudied. The lncRNA lncTCF7 has been shown to promote tumor progression by recruiting the SWI/SNF complex to the TCF7 promoter, activating its expression and the WNT signaling pathway. However, how lncTCF7 recruits SWI/SNF remains to be determined, and lncTCF7-specific binding partners are unknown. Using RNA-pulldown and quantitative mass spectrometry, we identified a novel interacting protein partner for lncTCF7, SND1, a multifunctional RNA binding protein that can also function as a transcription co-activator. Knockdown analysis of lncTCF7 and SND1 reveals that they are both required for the expression of TCF7. Chromatin immunoprecipitation assays suggest that both SND1 and lncTCF7 are required for recruiting the SWI/SNF chromatin remodeling complex, and these functions, in tandem, activate the expression of TCF7. Finally, using structural probing and RNA-pulldown of lncTCF7 and its subdomains, we highlight the potential binding region for SND1 in the 3’-end of lncTCF7. Overall, this study highlights the critical roles lncRNAs play in regulating gene expression and provides new insights into the complex network of interactions that underlie this process.

## Introduction

Long non-coding RNAs (lncRNAs) are RNA transcripts with no protein-coding potential, are longer than 200 nucleotides, and have recently emerged as critical players in various biological processes^1–6^. LncRNAs have been implicated in multiple morbidities, including inflammation, cancer, and neurological diseases such as Alzheimer's and schizophrenia^7–10^. In cancer specifically, lncRNAs are characterized as drivers of cancer progression through several pathways and may serve as useful biomarkers and potential therapeutic targets^11^. Therefore, it remains essential to investigate the mechanisms behind lncRNA-based regulation of the cellular pathways.

For instance, lncRNAs regulate transcription via epigenetic pathways such as DNA methylation, histone modification, and chromatin remodeling^12,13^. Well-known lncRNAs, like HOTAIR and XIST, have functional relationships with the histone-modifying complex PRC2^14^. However, recent interactome studies of both Xist and HOTAIR have revealed novel protein partners that mediate the recruitment and activity with PRC2^15–17^. Emerging studies show that lncRNAs also interact with the SWI/SNF (SWItch/Sucrose Non-Fermentable) chromatin remodeling complex, although the functional relationships are much less defined^18^. The number of characterized lncRNAs that regulate transcription is growing, and the mechanisms behind these functions are still understudied.

One such example is the lncRNA lncTCF7, which is dysregulated in various cancers, including hepatocellular carcinoma (HCC), colorectal cancer (CRC), and glioblastoma (GBM)^10,19–22^. LncTCF7 promotes the expression of a neighbouring gene, WNT signaling factor TCF7, and drives tumor growth and cancer progression in mouse models^19^. By promoting TCF7 expression, lncTCF7 is reported to upregulate WNT signaling genes and stemness markers, thereby driving tumor progression. In HCC, evidence indicates that lncTCF7 recruits the SWI/SNF chromatin remodeling complex *in cis* to the TCF7 promoter to regulate its transcription^19^. However, the molecular details behind this recruitment process and the specific lncTCF7 binding partners are unknown.

In this study, we aim to elucidate the mechanism of lncTCF7’s role in transcription regulation. First, we validated the transcription regulation role of lncTCF7 in GBM. Next, we identified Staphylococcal Nuclease Domain-containing protein 1 (SND1) as a novel binding partner of lncTCF7 using RNA-pulldown assay and quantitative mass spectrometry. Finally, we characterized the functional role of lncTCF7-SND1 partnership using cellular and biochemical assays, and revealed that both are required for upregulating TCF7 expression. This study identifies a novel mechanism involving lncRNAs, SND1, and SWI/SNF complex in transcription regulation.

## Material and methods

### Cell culture

U251 (Sigma-Aldrich, Allentown, PA, USA, #09063001) and U87 (kind gift from the lab of Dr. Mauricio Reginato, Drexel University College of Medicine, Philadelphia, PA, USA) glioblastoma cells were cultured in Dulbecco’s Modified Eagle’s medium (DMEM; Thermo Fisher Scientific, Waltham, MA, USA #11995065) supplemented with 10% HyClone bovine growth serum (FBS; Cytiva, Marlborough, MA, USA #SH30541.03), 1% L-glutamine (Corning Life Sciences, Teterboro, NJ, USA #25005CI) and 1% penicillin/streptomycin (Genesee Scientific, San Diego, CA, USA #25-512).

### Constructs

Lentivirus packaging plasmids, pCMV-VSV-G, pRSV-Rev, and pMDLg-pRRE, were generously donated by Dr. Jane Clifford (Drexel University College of Medicine, Philadelphia, PA, USA). Custom-made lncTCF7 targeting shRNA lentiviral plasmids, along with the nontargeting scramble lentiviral plasmid, were purchased from Genecopoeia (Rockville, MD, USA). Following are the target sequences: shlncTCF7-1 (GCTGAACTTTGGATAAGATCT) and shlncTCF7-2 (GGTCATGGGCAACAACCAAAT).

### Lentivirus production

HEK-293 T cells were transfected with 6 μg of expression plasmid and 2 μg each of the packaging plasmids following the manufacturer’s protocol of GeneJet transfection reagent (SignaGen, Fredrick, MD, USA #110001). The virus was collected 48 h post-transfection and stored at − 80 °C with 8 μg/mL polybrene (AmericanBio, Canton, MA, USA #AB01643). Stable knockdown or control cell lines were generated by infection with the respective lentivirus, followed by selection with puromycin.

### siRNA-mediated knockdown analysis

Silencer siRNAs targeting SND1 and FAM-labeled negative control were purchased from Thermo Fisher Scientific (Waltham, MA, USA # S25749). 20 μM siRNA was transfected into U251 cells using the Lipofectamine RNAiMax transfection reagent (Thermo Fisher Scientific, Waltham, MA, USA #12778100). Cell media was changed 6 and 16 h post-transfection. Cells were collected for analysis 48 h after transfection.

### Cell lysate for biotin RNA-pulldown

U251 cells were grown to 80% confluency on 15 cm plates. Cells were washed with PBS, detached using a cell scraper, pelleted, and flash-frozen. Cell pellets were stored at − 80 °C. Nuclear extraction was performed following the NEXSON protocol, with minor modifications^23^. Briefly, cell pellets were resuspended in Nuclear Lysis Buffer I (10 mM HEPES pH 7.4, 20 mM KCl, 1.5 mM MgCl_2_, 0.5 mM EDTA, 1 mM TCEP, Halt Protease Inhibitor Cocktail (Thermo Fisher Scientific, Waltham, MA, USA #87786)), and RNaseOUT (Thermo Fisher Scientific, Waltham, MA, USA #10777019)^24^. The lysate was sonicated with six cycles (10 s on, 30 s off) using the Bioruptor Pico (Diagenode, Denville, NJ, USA). Nuclei were collected by centrifugation and resuspended in Nuclear Lysis Buffer II (20 mM Tris-HCl pH 7.5, 50 mM KCl, 1.5 mM MgCl_2_, 2 mM TCEP, 0.4% sodium deoxycholate, 1.0% n-dodecyl-D-maltoside, 0.1% N-lauroylsarcosine (NLS)). The nuclear lysate was treated with 200X DNAse Salt Solution (500 mM MgCl_2_, 100 mM CaCl_2_) and 20 U TurboDNAse (Invitrogen, Waltham, MA, USA #AM2238) for 12 min at 37 °C, and the insoluble fraction was removed by centrifugation (16,000 g, 10 min, 4 °C). The protein concentration was determined using Pierce 660 nm Protein Assay (Thermo Fisher Scientific, Waltham, MA, USA, #22660), and the nuclear lysate was flash-frozen and stored at − 80 °C.

### Biotinylated RNA in vitro transcription

In vitro transcription of biotinylated RNA was performed following previously published protocols, with an addition of biotin-14-CTP (Thermo Fisher Scientific, Waltham, MA, USA #19519016) in a ratio of 1:9 biotin-rCTP to normal rCTP^25–27^. The incorporation of biotin-rCTP was quantified using QuantTag Biotin Quantification Kit (Vector Laboratories, Newark, CA, USA #BDK-2000).

### Biotinylated RNA-pulldown

The pulldown of biotinylated RNA and associated cellular proteins was performed as previously described with few modifications^27^. Briefly, 500 μg protein from nuclear lysate was precleared with 125 μL washed streptavidin magnetic beads (New England Biolabs, Ipswich, MA, USA #S1420S) for 45 min at room temperature with agitation. While pre-clearing the lysate, the biotinylated RNA was refolded in folding buffer (25 mM HEPES pH 7.4, 150 mM KCl, 1 mM EDTA) by heating to 90 °C for 2 min, then placed on ice for 1 min. This was followed by an addition of MgCl_2_ to a final concentration of 12 mM MgCl_2_ for a final incubation at 37 °C for 30 min. Next, 30 pmol of refolded RNA was added to each sample, and binding occurred for 45 min at room temperature with agitation. This was followed by adding 125 μL washed streptavidin magnetic beads for 30 min at room temperature with agitation. Beads were washed two times with Nuclear Lysis Buffer II and four times with 10 mM Tris-HCl pH 7.4 with incubation for five minutes at room temperature with agitation between each wash. Beads were thoroughly dried on the magnetic rack and stored at − 80 °C.

### Quantitative mass spectrometry analysis

The Center for Advanced Biotechnology and Medicine (Rutgers University, NJ, USA) performed on-bead digestion, mass spectrometry, and database search. On-beads digestion and mass spectrometry were performed as previously described^28^. For the database search, the peak list of the LC–MS/MS was generated by Thermo Proteome Discoverer (v. 2.4) into MASCOT Generic Format (MGF) and searched against UniProt human reference proteome database, plus a database composed of common lab contaminants using in house version of X!Tandem (GPM Furry)^29^. Other common contaminants were removed based on published common contaminants^30^. Search parameters were as follows: fragment mass error: 20 ppm, parent mass error: +/− 7 ppm; fixed modification: none; flexible modifications: Oxidation on Methionine; protease specificity: trypsin (C-terminal of R/K unless followed by P), with 1 miss-cut at preliminary search and 5 miss-cut during refinement. Only spectra with log < − 2 were included in the final report, and protein species with fewer than 2 peptides were removed. To account for any non-RNA background interactions, an enrichment of log_2_ ≥ 3.5 (fold enrichment 11.3) was applied^16^. To define lncTCF7 interactions, proteins were ranked by peptide abundance. Interacting partners for each experiment were defined as at least twice as abundant in the experimental sample over the control sample.

### Western blot analysis

All the antibodies used in this study are listed in Supplementary Table S1. Protein concentration was measured using Pierce 660 nm Protein Assay (Thermo Fisher Scientific, Waltham, MA, USA, #22660). The samples were diluted in Laemelli sample buffer (3X, 150 mM Tris-HCl pH 6.8, 6% SDS, 6 mM EDTA, 30% glycerol, 0.01% Bromophenol blue) and boiled for 10 min at 95 °C. The proteins were separated on SDS-PAGE gels (4–15% TGX Minigels, BioRad, Hercules, CA, USA #4561081), transferred to a nitrocellulose membrane, blocked with 5% dry milk in PBST (1X PBS, 0.1% Tween-20), before incubating with primary antibodies (Supplementary Table S1). Immunodetection was performed using SuperSignal West Pico PLUS Chemiluminescent Substrate (Thermo Fisher Scientific, Waltham, MA, USA #34580) and the GeneSys G: Box F3 gel imaging system (Syngene). Band intensities were quantified using ImageJ, normalized to loading control, and the values are indicated below the blot^31^. All the raw western blot images are provided in the supplementary information.

### RNA immunoprecipitation

U251 and U87 cells were grown to 80% confluency on 15 cm plates. RNA crosslinking was performed using 0.1% formaldehyde in PBS for 10 min at room temperature, followed by adding 125 mM glycine to quench. Nuclear extraction was performed following the NEXSON protocol, with minor modifications^23^. Nuclear pellets were resuspended in IP Dilution Buffer from Thermo Fisher Scientific (Waltham, MA, USA #88804). Antibody binding was performed overnight at 4 °C with corresponding antibodies (Supplementary Table S1), and antibody capture was performed for 2 h at 4 °C with Pierce ChIP-grade Protein A/G magnetic beads (Thermo Fisher Scientific, Waltham, MA, USA #88804). Beads were washed with IP Wash Buffer (Thermo Fisher Scientific, Waltham, MA, USA #88804) and eluted using IP Elution Buffer (Thermo Fisher Scientific, Waltham, MA, USA #88804) for 30 min at 65 °C. The cross-linking of both input and elution samples was reversed using proteinase K treatment at 65 °C for 1.5 h. RNA was purified using the manufacturer’s protocol for TRIzol-LS reagent (Thermo Fisher Scientific, Waltham, MA, USA #10296028). RNA elution and input were treated with TurboDNAse (Invitrogen, Waltham, MA, USA #AM2238) and concentrated using Zymo RNA Clean & Concentrate Kit (Zymo, Irvine, CA, USA #R1015). Samples were taken through reverse transcription using SSIII First-Strand Synthesis (Thermo Fisher Scientific, Waltham, MA, USA, #18080051) and qPCR using SsoAdvanced Universal SYBR Green Supermix (BioRad, Hercules, CA, USA #1725270), as described below. Percent input analysis was performed using a modified ∆∆C_q_ method in which all samples were normalized to input RNA levels.

### Co-immunoprecipitation

Co-immunoprecipitation (Co-IP) was performed by using the Pierce Classic Magnetic IP/Co-IP kit (Thermo Fisher Scientific, Waltham, MA, USA #88804). Briefly, total U251 cell lysates were lysed and collected in Pierce IP Lysis/Wash Buffer (0.025 M Tris pH 7.4, 0.15 M NaCl, 0.001 M EDTA, 1% NP-40, 5% glycerol) containing 1X protease inhibitors (Roche Applied Science, Indianapolis, IN, USA #04693124001). Protein concentrations of total cell lysates were determined using Pierce BCA Protein Assay Kit (Thermo Fisher Scientific, Waltham, MA, USA #23227). Ten percent of the total cell lysates were collected separately and used for input. Cell lysates were incubated with 10 µg of anti-SND1 as the IP antibody (Proteintech, Rosemont, IL, USA #10760-1-AP) overnight at 4 °C. 10 µg of anti-rabbit IgG (Cell Signaling Technology, Beverly, MA, USA #7074S) was used as a negative control. After incubating the antigen/antibody complexes with pre-washed magnetic beads for 1 h at room temperature, beads were washed with washing buffer and ultra-pure water. The antigen/antibody complexes were then eluted in Lane Marker Sample Buffer (0.06 M Tris-HCl pH 6.8, 1% SDS, 10% glycerol, lane marker tracking dye) with 50 mM DTT via incubation for 10 min at room temperature. The precipitates were subjected to a western blot with BRG1 as the primary antibody (IB) (Cell Signaling Technology, Beverly, MA, USA #52251). All antibodies used for Co-IP are listed in Supplementary Table S1.

### Chromatin immunoprecipitation

Cell fixation and chromatin preparation were performed as described by Swift et al.^32^. Chromatin immunoprecipitation followed the Pierce Magnetic ChIP protocol (Thermo Fisher Scientific, Waltham, MA, USA #26157). Briefly, immunoprecipitated genomic DNA was incubated with each appropriate antibody (Supplementary Table S1) overnight at 4 °C with agitation. Each IP reaction was incubated for 2 h at 4 °C with the Pierce ChIP-grade Protein A/G magnetic beads (Thermo Fisher Scientific, Waltham, MA, USA #26157). Beads were washed 4 times with 5 min incubations at room temperature with agitation between each wash. Immunoprecipitated DNA was eluted at 65 °C for 30 min with vigorous shaking, and the cross-linking of both input and elution samples was reversed using proteinase K treatment at 65 °C for 1.5 h. DNA was cleaned and concentrated using DNA Clean & Concentrate Kit (Zymo, Irvine, CA, USA #D4013). ChIP-qPCR was performed via SsoAdvanced Universal SYBR Green Supermix (BioRad, Hercules, CA, USA #1725270) with respective primers (Supplementary Table S2)^19^. Human Negative Control Primer Set 1 (Active Motif, Carlsbad, CA, USA #71001) was used to evaluate the quality of ChIP samples. Fold enrichment of binding was calculated using a modified ∆∆C_q_ method in which all samples were normalized to the differences in input DNA.

### RT-qPCR

All the primers used for qPCR analysis are listed in Supplementary Table S2. Total RNA was extracted from cells using TRIzol reagent (Thermo Fisher Scientific, Waltham, MA, USA #15596026) following the manufacturer’s instructions. cDNA synthesis was performed using iScript Advanced cDNA Synthesis Kit (BioRad, Hercules, CA, USA #1725037). For RT-qPCR, PCR was performed using SsoAdvanced Universal SYBR Green Supermix (BioRad, Hercules, CA, USA #1725270) and a Bio-Rad CFX-96 real-time PCR detection system. Target genes were normalized to GAPDH levels in each sample. The relative gene expression levels and fold change were determined using the 2^(−ΔΔCt) method.

### Bioinformatics analysis

Expression of SND1 across TCGA data was examined using the online web server GEPIA2^33^. The GEPIA2 survival analysis tool was employed to perform the Kaplan–Meier analysis, and the correlation analysis tool was used to determine the correlation between the expression of SND1 and TCF7 in the GBM tumor sample.

### Statistical analysis

Unless otherwise noted in the figure legend, experiments were performed in biological triplicate, with each replicate a representative mean of 2–3 technical replicates. Where appropriate, data is represented as the mean and standard error of the mean (S.E.M). Significant differences between groups were determined using the ratio Student’s t-test. Where statistical significance is not identified, data is assumed to have a *p*-value greater than 0.05.

## Results

### Validation of TCF7 transcription regulation by lncTCF7 in GBM

**Figure 1:**
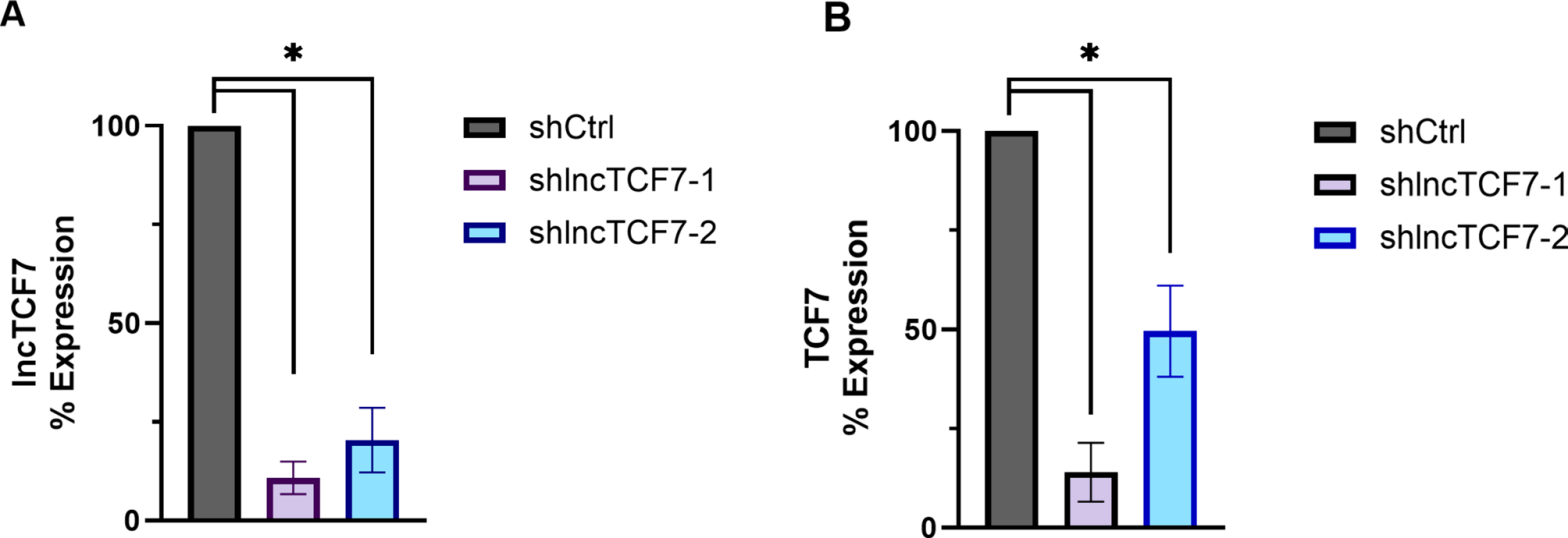
Stable knockdown of lncTCF7 validated its role in TCF7 transcription regulation. (**A**) qRT-PCR analysis of lncTCF7 knockdown by two independent, stably expressed shRNAs in U251 cells. (**B**) WNT signaling pathway protein TCF7 expression loss, analyzed by qRT-PCR. Data are shown as average relative knockdown ± SEM (two-tailed ratio t-test, n = 3, *P-value < 0.05).

When we began this study on lncTCF7, its function had been only briefly characterized in a handful of other publications^19–22^. Therefore, we first aimed to validate its proposed functional role. The best-characterized function of lncTCF7 is regulating the expression of protein TCF7. In multiple cancers, lncTCF7 knockdown resulted in the loss of TCF7 expression, which downregulated the WNT signaling pathway^19–22^. To validate the role of lncTCF7 in regulating TCF7 expression, we stably knocked down lncTCF7 in the U251 glioblastoma cell line using two shRNA plasmids (Fig. 1A). Cells expressing scramble shRNAs were used as controls. As expected, knocking down lncTCF7 decreased the expression of TCF7, as analyzed by qRT-PCR (Fig. 1B).

### Identifying novel interacting partners of lncTCF7

Recent studies utilizing mass spectrometry have surveyed the interactomes of multiple lncRNAs and have uncovered several unique, novel interacting partners for each lncRNA^15–17^. Many RNA binding proteins may interact indiscriminately with lncRNAs, thereby directly impacting their function or indirectly facilitating a functional outcome by affecting lncRNA localization, stability, or multiple factors simultaneously. Identifying the specific protein partners of a given lncRNA is imperative to gain insights into its function. Therefore, we set out to identify specific protein partners of lncTCF7.

**Figure 2:**
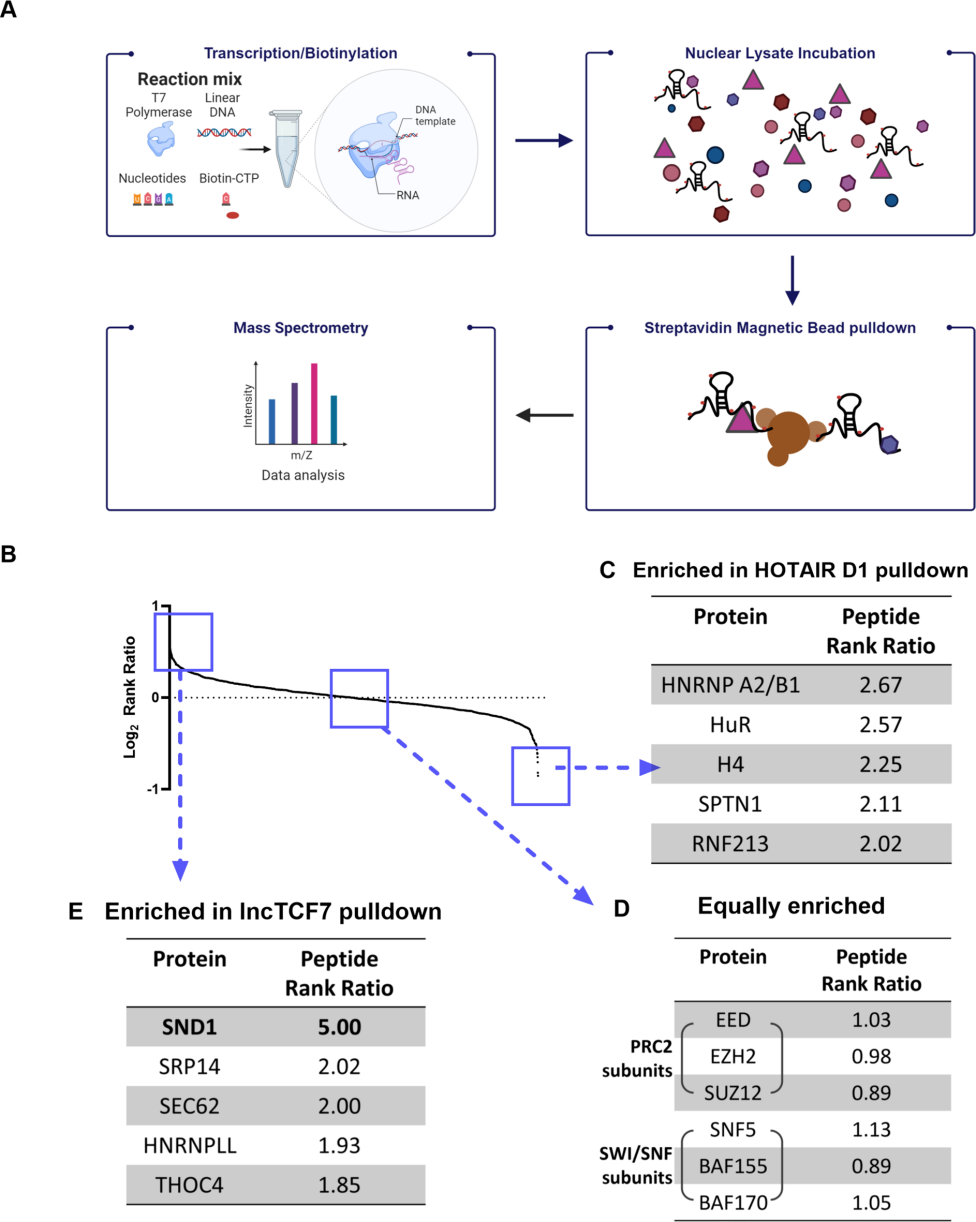
RNA affinity pulldown and mass spectrometry identify novel binding partners of lncTCF7. (**A**) Schematic of biotinylated RNA-pulldown and mass spectrometry method. (**B**) The enriched proteins, ordered based on log 2 rank ratio. (**C**) The top enriched proteins in HOTAIR D1 pulldown identify two previously studied HOTAIR functional partners, HNRNP A2/B1 and HuR. (**D**) Proteins similarly enriched in both lncTCF7 and HOTAIR D1 pulldowns. (**E**) The top five proteins enriched in the full-length lncTCF7 pulldown (data averaged of two replicates).

We utilized a biotinylated RNA-pulldown assay to identify partners that may facilitate lncTCF7 function (Fig. 2A). This method enriches potential functional protein partners by incubating cellular lysate with biotinylated RNA and characterizing the pulldown products via mass spectrometry^27^. Because the RNA probes are made in-house, this method affords control over the biotinylation, concentration, and folding of the RNA. As a control, we used the lncRNA HOTAIR domain 1 (HOTAIR D1) in parallel to the lncTCF7 pulldown as it is similarly sized to lncTCF7 and, importantly, its binding partners are well-characterized^15^. Therefore, we expect that promiscuous RNA binding proteins will interact similarly with both lncRNAs and that known HOTAIR D1 binding partners will be preferentially enriched in the HOTAIR D1 sample. Consequently, the protein species uniquely enriched in the lncTCF7 sample would represent potential lncTCF7-specific interacting partners.

After removing common mass spectrometry contaminants and proteins not significantly enriched over the background, the remaining proteins were analyzed similarly to previously published RNA-pulldown methods^30,34^. The mass spectrometry data were highly correlated between replicates (R > 0.99), and replicates were averaged (Suppl. Figure 1A and B). Proteins that ranked at least 1.5 times higher in the lncTCF7 sample than in the HOTAIR D1 control were identified as lncTCF7 enriched (Fig. 2B). Similarly, HOTAIR D1-enriched proteins were at least 1.5 times as highly ranked in HOTAIR D1 than in lncTCF7 (Supplementary Table S3)^16^. To test the validity of this method, we first investigated the highly enriched proteins in the HOTAIR D1 control. The most enriched protein in the HOTAIR D1 sample was HNRNP A2/B1, and the second most enriched protein was HuR (Fig. 2C). Importantly, both HNRNP A2/B1 and HuR have been identified as functional interacting partners of HOTAIR^15,35^. This indicates that the in vitro RNA-pulldown assay and data analysis are robust and reproducible, and the results are consistent with the literature.

Previous studies identified three SWI/SNF core subunits, SNF5, BRG1, and BAF170, as interacting partners of lncTCF7^19^. These SWI/SNF subunits were equally abundant in lncTCF7 and HOTAIR D1 pulldowns (Fig. 2D). The abundance of SWI/SNF subunits in both lncTCF7 and HOTAIR pulldowns may either indicate that the SWI/SNF complex functions with both lncTCF7 and HOTAIR or support that the SWI/SNF subunits bind RNA promiscuously. In fact, previous in vitro binding studies indicated that the BRG1 and other SWI/SNF subunits promiscuously bind RNA^36^. In either scenario, it is likely that other proteins are more closely associated with lncTCF7 and are responsible for facilitating a more specific and functional interaction with the SWI/SNF complex. This possibility aligns well with other lncRNAs that regulate the SWI/SNF complex by binding non-SWI/SNF chromatin-associated proteins to recruit the complex^37,38^. Therefore, we examined proteins specifically enriched in the lncTCF7 sample. Notably, staphylococcal nuclease domain-containing protein 1 (SND1) was highly enriched in the lncTCF7 pulldown (Fig. 2E), indicating SND1 is a specific interacting partner of lncTCF7.

### RIP assay confirms that endogenous lncTCF7 binds SND1

**Figure 3:**
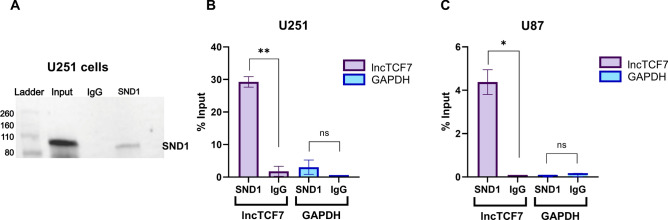
RIP analysis confirms that lncTCF7 interacts with SND1. (**A**) Western blot analysis demonstrates successful IP of SND1. (**B**) Recovery of lncTCF7 and GAPDH RNA, assessed by RT-qPCR following RIP of SND1 versus an IgG control in U251 cells. The graph depicts the average recovery ± SEM (two-tailed ratio t-test, n = 4). (**C**) Recovery of lncTCF7 and GAPDH RNA, assessed by RT-qPCR following RIP of SND1 versus an IgG control in U87 cells. The graph depicts the average recovery ± SEM (two-tailed ratio t-test, n = 3). *P-value < 0.05, **P-value < 0.01, ns implies not significant.

To validate these in vitro pulldown results in a physiologically relevant context, we utilized an orthogonal method of formaldehyde crosslinking RNA immunoprecipitation (f-RIP) in two glioblastoma cell lines. We first confirmed the protein IP by western blot analysis (Fig. 3A) and then investigated the recovery of RNA in both SND1 and IgG IPs using qRT-PCR analysis. The SND1 IP recovered significantly more lncTCF7 than the control IgG in U251 cells. As a control, we also tested for the recovery of GAPDH mRNA and noticed no recovery in either SND1 or IgG IPs (Fig. 3B). To add robustness and ensure this interaction was not cell-line specific, we repeated the f-RIP experiment in U87 glioblastoma cells and found similar results (Fig. 3C). These results confirm that endogenous lncTCF7 binds SND1, and this interaction is present in the two different glioblastoma cell lines.

### SND1 regulates the expression of TCF7

We next sought to characterize the functional relationship between SND1 and TCF7 expression. SND1 is a multifunctional RNA binding protein that functions as a transcription co-activator, interacts with chromatin remodeling complexes, and modulates mRNA decay pathways^39–41^. Intriguingly, many of these functions are in tandem with lncRNAs. Previous literature shows that lncRNAs regulate the activity of SND1 by recruiting it to specific promoter regions^41^. For example, in breast cancer, linc00668 recruits SND1 to the promoters of stemness factors, while during limb development, lncRNA-HIT recruits SND1 in complex with CREB-binding protein (CBP) to regulate the transcription of pro-chondrogenic genes^41,42^. More globally, SND1 accumulates at UV-induced DNA damage sites and recruits histone modifiers such as the SWI/SNF chromatin remodeling complex^43^. Based on the previous literature on SND1, we therefore hypothesized that the lncTCF7-SND1 interaction may play a functional role in regulating gene expression.

**Figure 4:**
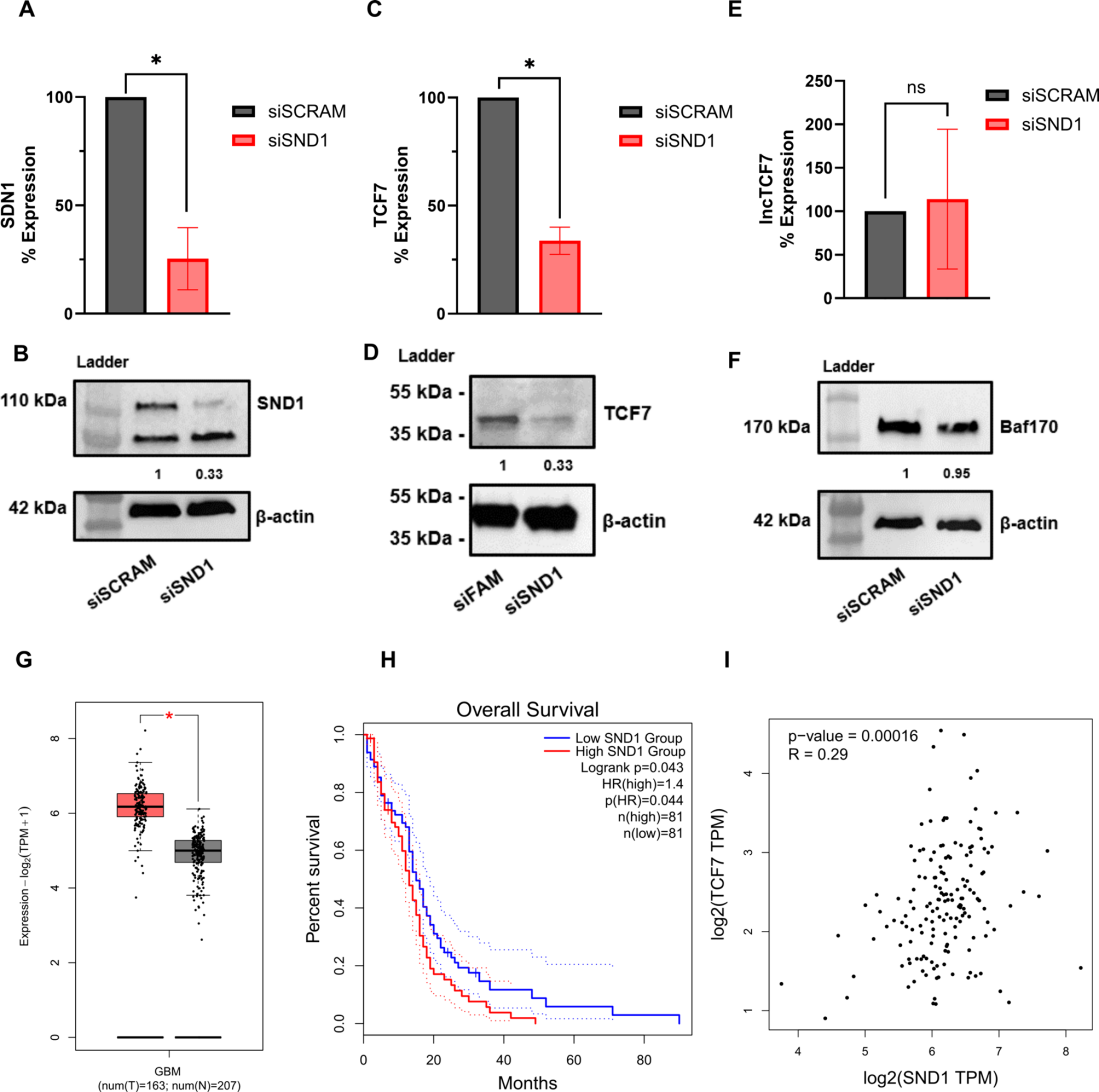
SND1 is required for TCF7 expression and relates to poor patient prognosis in glioblastoma. (**A**) qRT-PCR analysis of SND1 knockdown by transient siRNA transfection in U251 cells. (**B**) Confirmation of SND1 knockdown by western blot analysis (representative data from duplicate experiments). (**C**) SND1 knockdown causes loss of WNT signaling pathway protein TCF7 expression at the RNA level, analyzed by qRT-PCR. (**D**) SDN1 knockdown causes loss of TCF7 expression at the protein level, as analyzed by western blots (representative data from triplicate experiments). (**E**) SND1 knockdown does not affect lncTCF7 expression. (**F**) Western blot analysis shows no change of BAF170 in SND1 knockdown cell lines (representative data from duplicate experiments). (**G**) Gene expression analysis of SND1 in GBM tissues (red) versus in nontumor tissues (grey). (**H**) Overall survival plot of GBM patients depending on the level of SND1 expression. (**I**) Correlation analysis between the expression of SND1 and TCF7 in GBM. For all qRT-PCR experiments, data are shown as average relative knockdown ± SEM (two-tailed ratio t-test, n = 3). *P-value < 0.05, **P-value < 0.01, ns implies not significant. For all western blots, band intensities were quantified and normalized to the loading control. Values are shown below the blot.

To characterize the lncTCF7-SND1 interaction, we first investigate the impact of SND1 on lncTCF7’s previously characterized function in regulating TCF7 expression and SWI/SNF localization. In U251 cells, we silenced SND1 expression using siRNAs, and confirmed the knockdown using RT-qPCR (Fig. 4A) and western blot (Fig. 4B). Knocking down SND1 also downregulated the expression of TCF7 at both RNA (Fig. 4C) and protein levels (Fig. 4D). At the same time, knocking down SND1 did not impact the expression of lncTCF7 (Fig. 4E) and BAF170 (Fig. 4F). These results suggest that SND1 regulates the expression of TCF7 but not lncTCF7 and BAF170.

To explore the validity of these results and the role of SND1 in glioblastoma (GBM), we performed bioinformatics analysis on the cancer genome atlas (TCGA) datasets available in the GEPIA2 database^33^. Gene expression analysis across GBM patient samples revealed that SND1 is highly overexpressed in GBM (Fig. 4G). Further, GBM patients with high-level expression of SND1 showed a decreased overall survival rate (Fig. 4H). Finally, we found a positive correlation between the expression level of SND1 and TCF7 in GBM (Fig. 4I). These results indicate that SND1 regulates the expression of TCF7 in GBM.

### lncTCF7-SND1 interaction recruits SWI/SNF complexes to the TCF7 promoter

**Figure 5:**
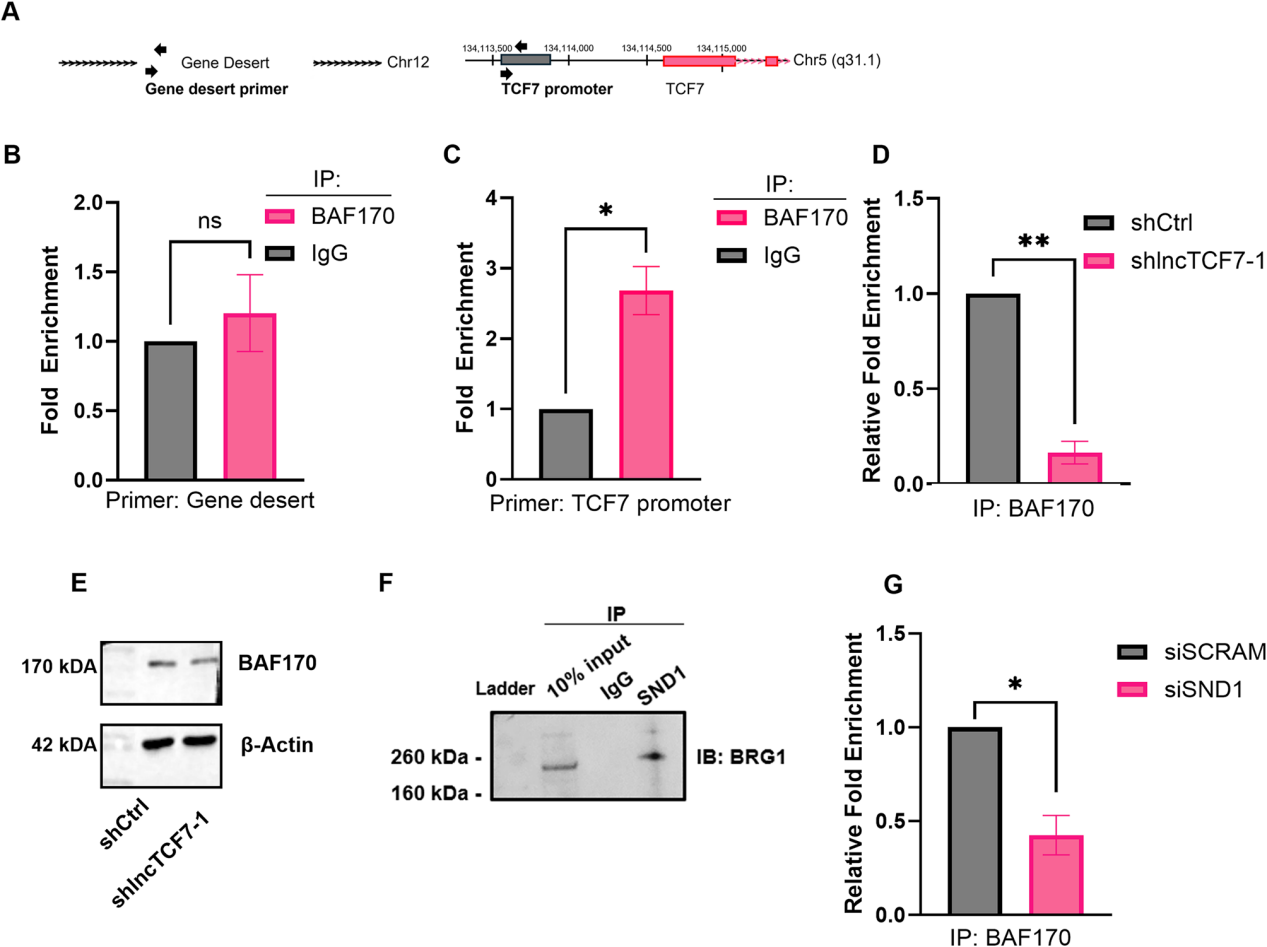
SND1 and lncTCF7 impact the localization of chromatin factors to its promoter. (**A**) The binding sites of the primers used in chromatin immunoprecipitation experiments. (**B**) No enrichment of the SWI/SNF core subunit BAF170 via chromatin immunoprecipitation (ChIP) at the gene desert on human chromosome 12, validating the quality of our ChIP samples. Graphs show qPCR analysis of average enrichment over IgG control ± SEM (two-tailed ratio t-test, n = 3). (**C**) Fold enrichment of BAF170 via ChIP at the TCF7 promoter (two-tailed ratio t-test, n = 3). (**D**) Upon knockdown of lncTCF7, BAF170 fails to localize to the TCF7 promoter. (two-tailed ratio t-test, n = 3). (**E**) Western blot analysis shows no change in the expression of BAF170 in lncTCF7 knockdown cell lines (representative data from duplicate experiments). Normalized values are shown below the blot. (**F**) Co-immunoprecipitation showed the interaction between SND1 and BRG1, the SWI/SNF core subunit. The band may have slightly shifted upwards due to the lack of a denaturation step in the protocol. (**G**) Upon SND1 knockdown, BAF170 fails to localize to the TCF7 promoter. Graphs show qPCR analysis of average relative enrichment over non-targeting control ± SEM (two-tailed ratio t-test, n = 3). *P-value < 0.05, **P-value < 0.01, ns implies not significant.

Next, we examined the ability of lncTCF7-SND1 interaction to recruit SWI/SNF complexes to the TCF7 promoter. Previous studies have shown that lncTCF7 recruits the SWI/SNF complex to the promoter region of TCF7, and knockdown of lncTCF7 results in a loss of SWI/SNF localization at this promoter. However, these studies were performed in hepatocellular carcinoma cell lines. Therefore, we validated these results in the U251 cell line using chromatin immunoprecipitation combined with qPCR (ChIP-qPCR). Figure 5A shows the schematic of gene location and primers used for the ChIP assay. Negative control primers amplifying a gene desert genomic region were used to validate the quality of antibodies employed in our ChIP experiments (Fig. 5A). As expected, negative control primers have no significant enrichment of BAF170 at the gene desert genomic region (Fig. 5B). Our ChIP experiments also confirmed the SWI/SNF subunit BAF170 enrichment at the previously reported TCF7 promoter region, which is 1500–1000 bp upstream of the TCF7 transcription start site (TSS) (Fig. 5C). Next, to determine the role of lncTCF7 in the localization of the SWI/SNF complex to the TCF7 promoter, we performed ChIP assays on lncTCF7 knockdown cells. Knockdown of lncTCF7 resulted in a significant loss of BAF170 at the TCF7 promoter (Fig. 5D). We also note that stably knocking down lncTCF7 did not impact the expression of the SWI/SNF subunit BAF170 (Fig. 5E). These results validate and support the previously reported mechanism through which lncTCF7 recruits the SWI/SNF complex to the TCF7 promoter and regulates its expression.

Next, we examined the role of SND1 in recruiting the SWI/SNF complex to the TCF7 promoter. Previous studies have identified SND1 as a transient interactor of the SWI/SNF complex (see supplementary data, Wolf et al.)^43,44^. However, these studies were conducted in different cell lines and under different conditions. To test if SND1 interacts with the SWI/SNF complex in GBM cell lines, we performed co-immunoprecipitation (Co-IP) assays in U251 cells. Our Co-IP results revealed that SND1 interacts with BRG1, a core SWI/SNF subunit (Fig. 5F). Together, our results show that SND1 interacts with the SWI/SNF complex and that the SWI/SNF complex localizes to the TCF7 promoter in glioblastoma cells (Fig. 5F and C). We then investigated the impact of SND1 on the SWI/SNF localization pattern. Interestingly, the knockdown of SND1 led to a notable loss of BAF170 at the TCF7 promoter (Fig. 5G). Thus, SND1 is required for the SWI/SNF complex to localize to the TCF7 promoter.

These results indicate that the partnership between SND1 and lncTCF7 plays a key role in regulating the localization of SWI/SNF subunits to the TCF7 promoter and activating TCF7 expression.

### The 3’-end of lncTCF7 is sufficient to pulldown SND1

Finally, we sought to characterize the regions of lncTCF7 which binds SND1. Previous literature showed, using RNA-pulldowns of lncTCF7 fragments, that the third exon of lncTCF7 was sufficient to pulldown three core SWI/SNF subunits (BAF170, SNF5, and BRG1)^19^. Additionally, our lab characterized the secondary structure of lncTCF7 using chemical probing and mutational profiling (SHAPE-MaP and DMS-MaP), which predicted that the native structure of lncTCF7 is over 50% based paired and that it folds into multiple subdomains that extend out of a central 4-way junction^45^. Our SHAPE-MaP secondary structure shotgun analysis identified that the third exon of lncTCF7 spanning nucleotides 468–683 (referred hereby as 3’-end domain) as an independent secondary structure domain (Suppl. Figure 2A).

Next, to examine if the 3’-end domain of lncTCF7 binds SND1, we performed affinity RNA-pulldown using the 3’-end domain as the bait, followed by mass spectrometry. Just as with the full-length lncTCF7, the 3’-end domain of lncTCF7 was biotinylated during transcription and folded before binding and pulldown. The correlations of mass spectrometry data between replicates for lncTCF7 exon 3 were > 0.99 (Suppl. Figure 1C). Interestingly, the lncTCF7 3’-end domain was sufficient to pulldown SND1, albeit less dramatically than for the full-length lncTCF7 (Suppl. Figure 2B). These results suggest that the 3’-end region of lncTCF7 is sufficient to bind and pulldown SND1. However, further studies are required to understand the specific molecular motifs involved in lncTCF7-SND1 interaction.

**Figure 6:**
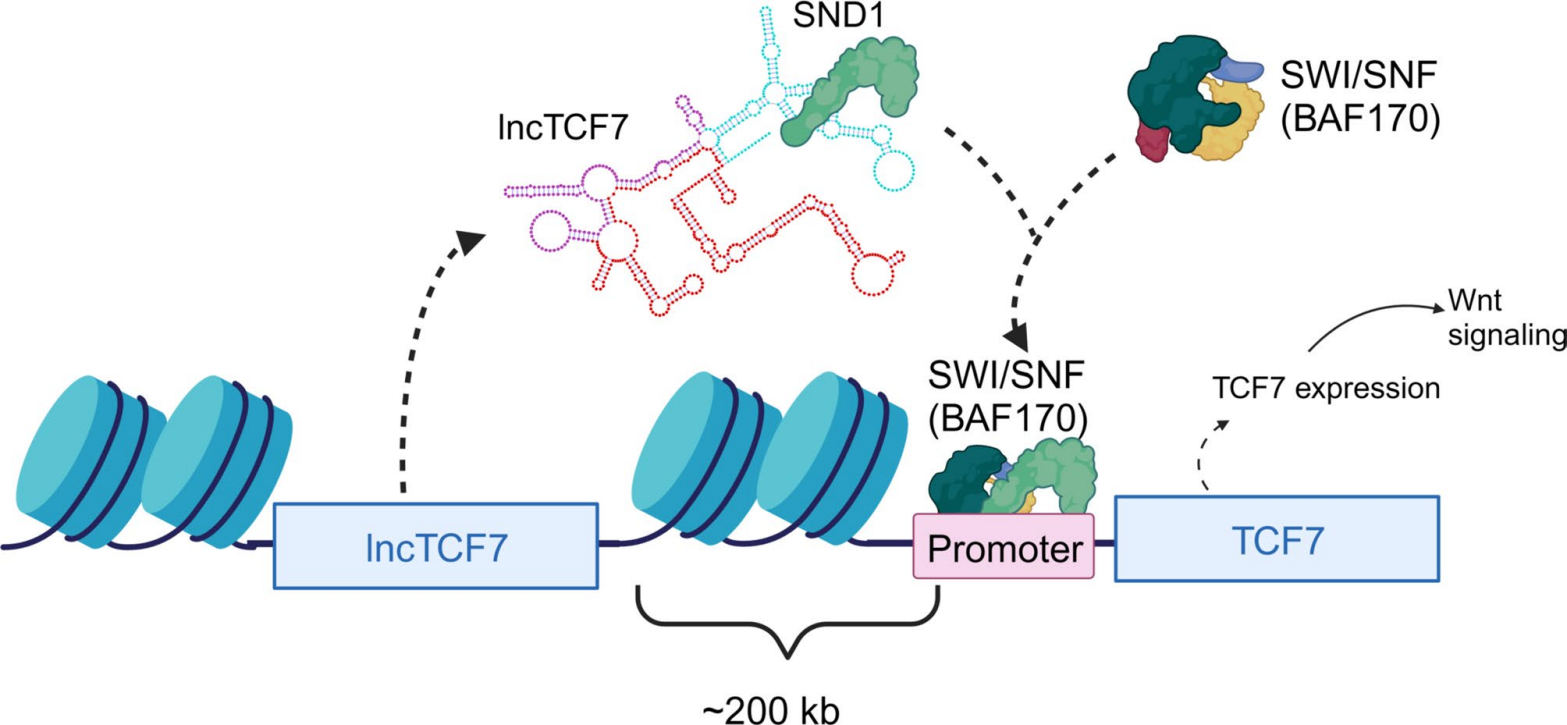
The proposed model of lncTCF7 and SND1’s regulation of TCF7 expression and SWI/SNF localization.

These findings imply a new model of lncTCF7 function in glioblastoma. Both lncTCF7 and SND1 regulate the expression of TCF7, and the localization of the SWI/SNF subunit to the TCF7 promoter depends on the molecular interaction between SND1 and lncTCF7 (Fig. 6).

## Discussion

This study employs biochemical and cellular assays to characterize the molecular function of a less studied lncRNA, lncTCF7, which recruits the SWI/SNF complex to the promoter region of the transcription factor 7 (TCF7) gene and regulates its transcription. Here, we identify a novel lncTCF7 protein partner, SND1, and investigate the functional role of this interaction. By knocking down either lncTCF7 or SND1, which ablated the SWI/SNF localization to the TCF7 promoter, we demonstrated that lncTCF7 regulates TCF7 transcription in glioblastoma cells through recruiting the SWI/SNF chromatin remodeling complex to its promoter region, and that SND1 is also required in this process. The 3`-end domain of lncTCF7 is sufficient to enrich for SND1, which highlights that this domain may serve in the mechanism of SND1 binding upstream of SWI/SNF recruitment and TCF7 transcription regulation. Taken together, this lays the groundwork for future investigations into the lncTCF7-SND1 functional interaction and its role in SWI/SNF recruitment.

### LncRNAs and the SWI/SNF complex

The SWI/SNF complex is an essential and highly conserved nucleosome-remodeling complex^46^. It is required for transcription regulation and critical for various cellular processes, including cell growth and differentiation^46^. LncRNAs associate with the SWI/SNF complex in humans^47^, mice^48^, and plants^49^, suggesting that non-coding RNAs may have an evolutionarily conserved role in SWI/SNF's function. LncRNAs modulate SWI/SNF function in various ways, including 1) recruiting the complex to a promoter, 2) competing with binding partners, or 3) altering the enzymatic activity of the complex^18,50,51^. For example, lncRNA Evf2 inhibits the enzymatic activity of the SWI/SNF core subunit BRG1, and lncTCF7 recruits the SWI/SNF complex to activate transcription, showcasing diverse lncRNA mechanisms in modulating the SWI/SNF function. Therefore, characterizing specific lncRNA interacting partners is crucial for comprehensively understanding their role in the SWI/SNF function. This work validates the role of lncTCF7 in recruiting the SWI/SNF complex to the TCF7 promoter in glioblastoma cells.

### Importance of identifying full interactome of a lncRNA

For other well-characterized lncRNAs, such as HOTAIR, recent publications have identified previously unknown and distinct interacting partners that facilitate the interaction with its more characterized functional partner, the PRC2 complex^52^. What was once thought to be direct interactions between PRC2 and HOTAIR is now known to require intermediary protein partners^52,53^. For example, HOTAIR functions with the PRC2 complex in histone methylation and gene repression, but since PRC2 chromatin localization is not specific, the specificity of the HOTAIR-regulated PRC2 localization is controlled and directed by other proteins such as HNRNP A2/B1^15,52^.

In this work, we employed RNA-pulldown followed by quantitative mass spectrometry to identify the interacting partners of lncTCF7, representing the first quantitative analysis of the lncRNA lncTCF7’s interactome. Recent studies have shown that chromatin-remodeling complexes may bind RNA promiscuously^14,36^. However, using another lncRNA as a control in pulldown experiments has been effective in eliminating promiscuous binders and identifying unique partners of RNAs^54^. Therefore, we used HOTAIR as a control in our biotinylated RNA-pulldown assays. We identified HOTAIR’s functional binding partner HNRNP A2/B1 as the most enriched protein in the HOTAIR pulldown, which indicates that our results on HOTAIR are consistent with the literature and therefore, our findings of lncTCF7 binding partners are robust^15^.

### Role of lncRNAs in SND1 function

Among the proteins strongly enriched in the lncTCF7 pulldown, SND1 is at the top of the list (Fig. 2B and Supplementary Table S3). Using an orthogonal technique f-RIP, we validated that the endogenous lncTCF7 binds SND1. We also ensured this interaction was not cell-line specific by repeating f-RIP in an additional GBM cell line. SND1 was initially identified as a transcription coactivator hijacked by the Epstein-Barr virus in lymphocytes to transcribe viral proteins^55^. SND1 is a multifunctional protein that modulates diverse pathways such as splicing, mRNA stability, and the formation of nuclear stress bodies^56–58^. RNA binding capacity is central to most of SND1’s identified functions. For example, in breast cancer, SND1 activates the expression of stemness factors by localizing to their promoters through the recruitment of linc00668^42^. Meanwhile, during limb development, lncRNA-HIT recruits SND1 in a complex with CREB-binding protein (CBP) to regulate gene expression associated with chondrogenesis^41^. SND1 also functions in complex with other proteins to impact gene expression and chromatin structures. In GBM, SND1 recruits histone acetyltransferase GCN5 to the RhoA promoter, forming a chromatin loop to upregulate RhoA expression^39^. Our findings contribute to the diverse functions of SND1, revealing its interaction with lncRNAs to modulate the SWI/SNF function.

### LncTCF7 and SND1 in glioblastoma

The interplay between SND1 and lncTCF7 in GBM may provide insight into broader mechanisms of gene regulation in cancer. Both SND1 and lncTCF7 are expressed at higher levels in GBM than in normal brain tissue, which correlates with worse patient outcomes^20^. Furthermore, overexpression of either SND1 or lncTCF7 in xenograft mouse studies induced tumor growth, highlighting their potential as cancer-driving factors^20,39^. This work represents the novel characterization of how lncTCF7 and SND1 interact to regulate the SWI/SNF chromatin remodeling complex, which is frequently mutated or dysregulated in cancers and neurodevelopmental disorders^59,60^. SND1 is an emerging drug target in cancer, and targeting protein–protein interaction sites in SND1, such as the interaction between SND1 and metadherin (MTDH), is an active area of research^61,62^. In this study, we characterized a novel interaction between lncTCF7-SND1, which may add to the pool of potential pockets that can be targeted with small molecules. Future studies will be focused on understanding the molecular motifs involved in this interaction.

### Supplementary Information

Supplementary Tables.

Supplementary Figures.

## Data Availability

The data underlying this article are available in the article and in its online supplementary material.
