# Supplementary material for: A novel partnership between lncTCF7 and SND1 regulates the expression of the TCF7 gene via recruitment of the SWI/SNF complex
Source: Sci Rep. 2024 Aug 21;14:19384. doi: 10.1038/s41598-024-69792-8 (PMC11339422; doi:10.1038/s41598-024-69792-8)
Supplement: Supplementary file 2 — Supplementary Figures. [file 41598_2024_69792_MOESM2_ESM.pdf]

Supplementary Figures for the manuscript

A novel partnership between lncTCF7 and SND1 regulates the expression of the TCF7 gene via recruitment of the SWI/SNF complex.

AUTHORS

Allison Yankey[1#], Mihyun Oh[1#], Bo Lim Lee[2], Tisha Kalpesh Desai[1] and Srinivas Somarowthu[3,*]

[1]Graduate Program in Molecular and Cell Biology and Genetics, Graduate School of Biomedical Sciences and Professional Studies, College of Medicine, Drexel University, Philadelphia, Pennsylvania, USA.

[2]Graduate Program in Biochemistry of Health and Disease, Graduate School of Biomedical Sciences and Professional Studies, College of Medicine, Drexel University, Philadelphia, Pennsylvania, USA.

[3]Department of Biochemistry and Molecular Biology, College of Medicine, Drexel University, Philadelphia, Pennsylvania, USA.

[#]These authors contributed equally.

*To whom correspondence should be addressed. Tel: 215-762-7133; Email: ss4473@drexel.edu

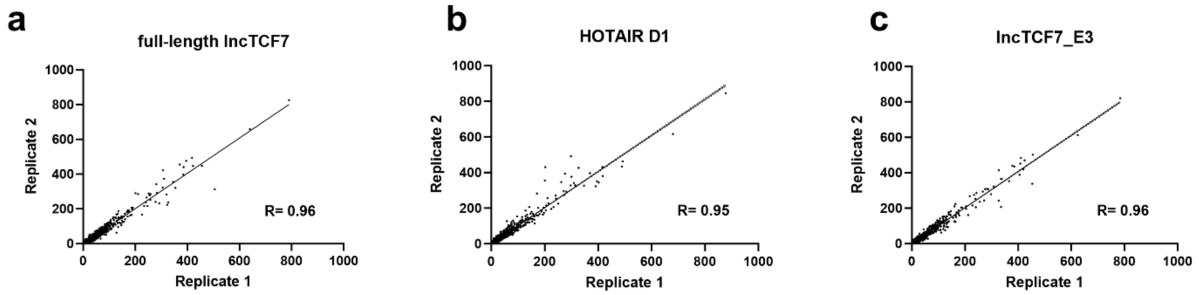

Supplementary Figure 1: Reproducibility of biotinylated RNA-pulldown and mass spectrometry. Both (A) full-length lncTCF7, (B) HOTAIR D1, and (C) 3’-end domain lncTCF7 fragment replicates show high correlation and reproducibility.

a

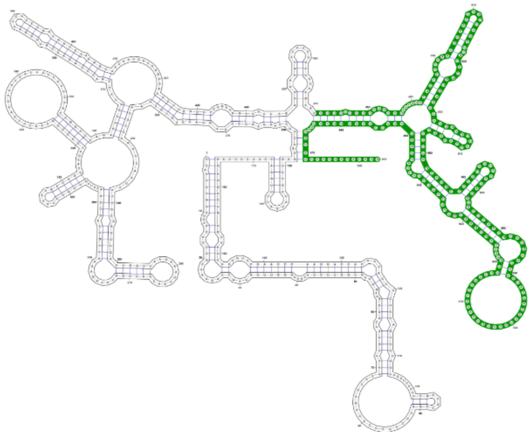

b

Enriched Rank	Protein	Peptide rank ratio
1	SND1	2.5
**2**	**MOES**	**2.33**
3	HnRNPLL	2.23
**4**	**THOC4**	**2.17**
5	GOGB1	2.03

Supplementary Figure 2: The structured 3'-end domain of lncTCF7 is sufficient for SND1 pulldown (A) The secondary structure of lncTCF7, 3'-end domain is colored in green. (B) The top enriched proteins of the 3'-end domain biotinylated RNA-pulldown.

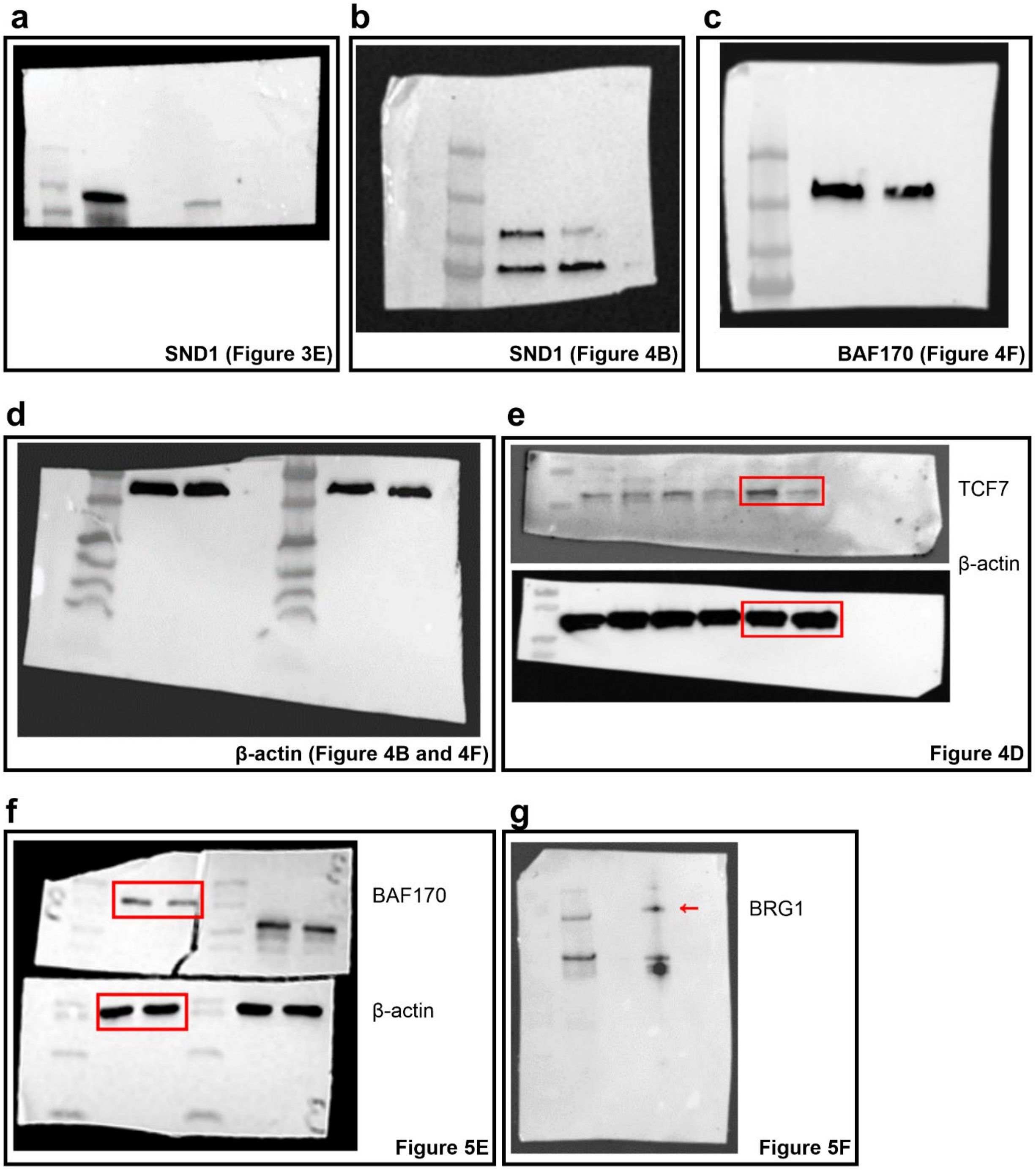

Supplementary Figure 3: Full raw images of western blots. Respective figures in the main text are indicated in each panel.
